# The wide base bipedicled (WIBB) flap in nipple-sparing skin-reducing mastectomy

**DOI:** 10.1038/s41598-024-52396-7

**Published:** 2024-04-22

**Authors:** Adriana Cordova, Matteo Rossi, Tiziana Roggio, Emanuele Cammarata, Calogero Cipolla, Salvatore Vieni, Francesca Toia

**Affiliations:** 1 Plastic and Reconstructive Surgery, Department of Surgical, Oncological and Oral Sciences (Di.Chir.On.S.), University of Palermo, Via del Vespro 129, 90127 Palermo, Italy; 2 St Andrew’s Centre for Plastic Surgery and Burns, Broomfield Hospital, Mid Essex Hospital Services NHS Trust, Chelmsford, Essex, UK; 3 Oncological Surgery Unit, Department of Surgical, Oncological and Oral Sciences (Di.Chir.On.S.), University of Palermo, Via del Vespro 129, 90127 Palermo, Italy

**Keywords:** Cancer, Surgical oncology

## Abstract

In this article, we present a modification of the NS/SRM technique in which the mastopexy design for skin reduction is undertaken with a wide-base bipedicled (WIBB) flap. The WIBB flap can be applied in both autologous and implant-based breast reconstruction. Our reconstructive algorithm is also presented. The clinical data of patients operated on from June 2017 to November 2022 were collected: 51 patients for a total of 71 breasts. Personal data, BMI, type and volume of implants used, and major and minor complications were analyzed by descriptive statistics. The mean age was 48.3 years. BMI ranged between 21.5 and 30.9 kg/m^2^. Thirty-one patients underwent unilateral mastectomy, while twenty patients underwent bilateral surgery. In 25 breasts, immediate reconstruction was performed with implants and ADM. In 40 breasts, reconstruction was performed with a subpectoral tissue expander, and in 6 breasts, reconstruction was performed with a DIEP flap. We observed only one case (1.4%) of periprosthetic infection requiring implant removal under general anesthesia. Minor complications occurred in 14.1% of patients. The use of both the WIBB flap and our algorithm maintained a low complication rate in our series, ensuring oncological radicality and a good aesthetic result at the same time.

## Introduction

Nipple-sparing mastectomy (NSM) is considered a safe oncological procedure for the treatment of selected noninvasive breast malignancies, such as ductal carcinoma in situ (DCIS), and for locally advanced invasive cancers without cutaneous or nipple involvement^1–3^. Recently, it has gained popularity as a risk-reducing procedure in BRCA mutation carriers^4–6^.

Although this technique permits oncological radicality and satisfactory cosmetic results^5,7,8^, it is not easy to perform on very large and ptotic breasts due to the presence of excess skin.

In this subset of patients, nipple-sparing skin-reducing mastectomy (NS/SRM) represents a suitable alternative^9,10^. However, it requires complex planning and a refined surgical technique^7,11^ to:

1. Determine the location and amount of skin to be excised
2. Safeguard the viability of the nipple areola complex and mastectomy flaps
3. Simultaneously plan the breast volume variation in immediate reconstruction cases

All these aspects make achieving good cosmetic results with NS/SRM more challenging.

In this paper, we present a modification of the NS/SRM technique in which the mastopexy design for skin reduction is performed with a wide base bipedicled (WIBB) flap and using our reconstructive algorithm.

The WIBB flap is a nipple-holding bipedicled flap that can be used both in immediate reconstruction (with autologous tissue or with implants) and in two-stage reconstruction with expanders. The choice of the reconstruction type (autologous or prosthetic, immediate or staged) depends on the tumor location, skin quality and thickness of the mastectomy flaps. The patient’s general health, physical characteristics and willingness were also taken into consideration.

## Methods

### Compliance with ethical standards

The study was performed in accordance with the principles stated in the World Medical Association Declaration of Helsinki. The study was approved by the local Ethical Committee of the University Hospital “Paolo Giaccone” of Palermo.

Informed consent was obtained from all participants of the study. Patients gave written informed consent to publish the information and the clinical pictures of their case in an online open access publication.

### Patient population and inclusion criteria

We applied our reconstructive protocol in 51 patients from June 2017 to November 2022. Patients aged < 18 years were excluded from the study.

Patients were selected based on oncological and reconstructive inclusion criteria.

The oncological and reconstructive inclusion criteria were as follows:

- Invasive carcinomas not amenable to breast conservative surgery (BCS) (multicentric disease, locally widespread disease or inflammatory breast cancer, presence of diffuse suspicious or malignant appearing microcalcifications on preoperative imaging, early pregnancy (RT is contraindicated during the I or II trimester), and mutations in BRCA1 and 2 genes).
- Absence of skin and/or nipple involvement
- BRCA-positive women who are candidates for risk-reducing mastectomy (RRM)

The reconstructive inclusion criteria were as follows:

- Medium-large and ptotic breasts
- Areola-inframammary fold (IMF) distance longer than 8 cm
- Sternal notch-nipple distance greater than 23 cm

Breast volume was assessed preoperatively: patients with medium-to-large breasts (a bra size greater than a C cup) were considered eligible for the technique.

Patient breasts were considered ptotic when the position of the nipple was below the inframammary fold. The degree of breast ptosis was calculated according to Regnault’s classification^12^: Grade I ptosis was diagnosed when the nipple was up to 1 cm below the crease. Grade II ptosis was defined when the nipple lay between 1 and 3 cm below the crease. Finally, Grade III ptosis was defined when the nipple position was more than 3 cm below the crease.

The volume to be reconstructed (implant size or flap volume) was determined with the aim of reconstructing a breast proportionate to the patient's conformation (implants between 380 and 600 cc were used) without necessarily respecting the weight and volume of the mastectomy specimen.

When indicated, the contralateral breast was symmetrized, with eventual mastopexy or breast reduction, during the same surgery.

Data regarding age, BMI, comorbidities, smoking status, BRCA mutations, cancer type, mastectomy (prophylactic/therapeutic), type and timing of reconstruction, implant size, complications (skin mastectomy flaps or nipple-areola complex necrosis, flap failure, implant exposure, infection, wound dehiscence, capsular contracture, seroma, hematoma) and length of follow-up were collected and analyzed by descriptive statistics. Major complications were defined as complications that resulted in implant loss and/or could not be managed conservatively and required additional surgical procedures under general anesthesia.

### Preoperative marking

**Figure 1:**
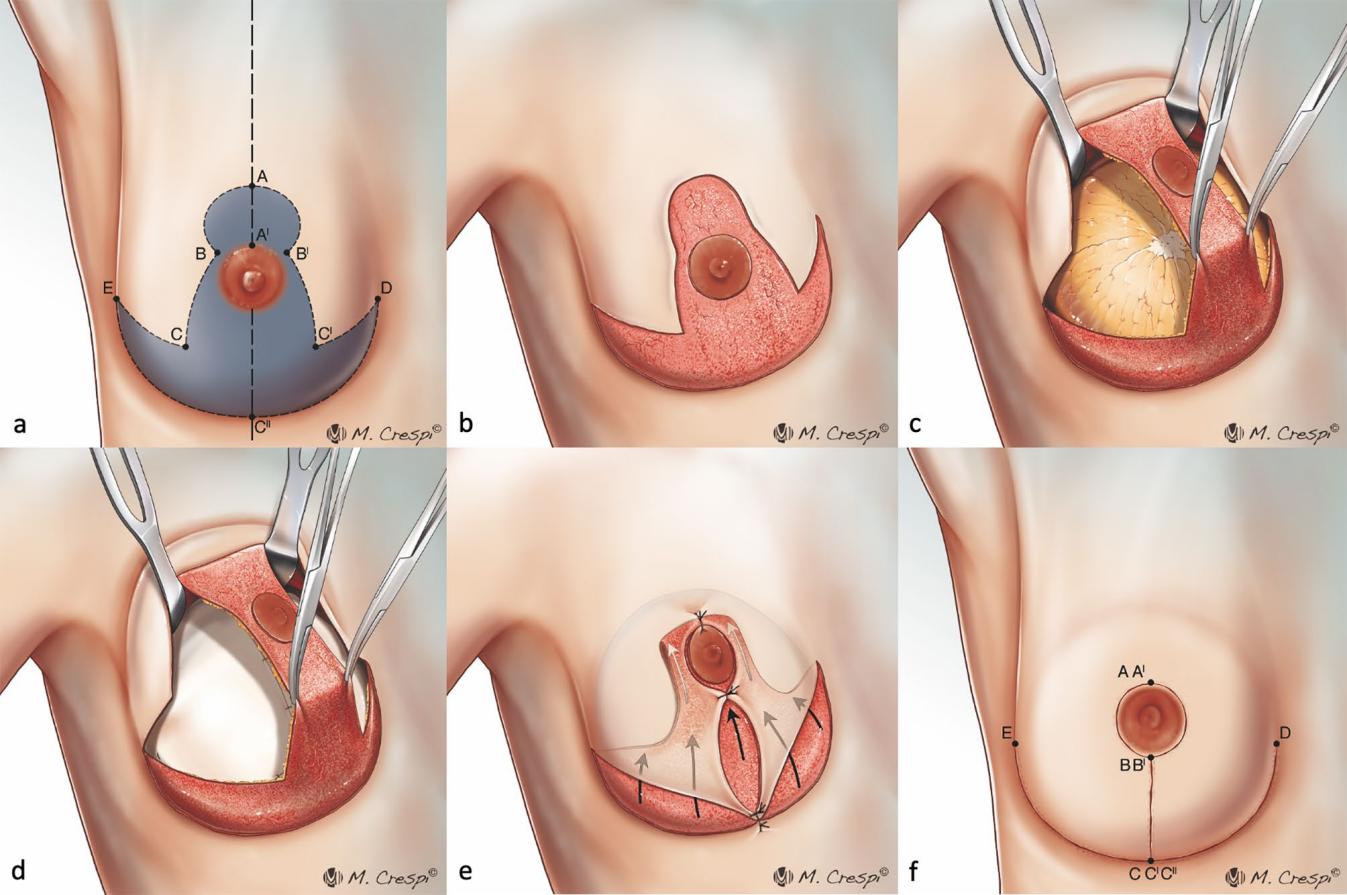
Illustration of surgical technique (**a**) Preoperative markings of modified SRM with NAC preservation (**b**) De-epithelialization of the entire marked area excluding the nipple-areola complex previously delimited with a cookie cutter of 38–45 mm in diameter (**c**) Incision along the medial and lateral aspects of the nipple-holding bipedicled dermal flap (WIBB flap), good exposure of breast tissue that is easily cleaved from the dermal flap upward and the pectoralis major muscle on the deep plane (**d**) The breast volume is replaced by means of one of the several reconstructive options and the new breast mound is secured under the dermal pedicle hosting the nipple-areola complex and the mastectomy flaps (**e**) Nipple-areola complex is sutured in the new position upward and skin flaps are approximated (**f**) Areola is sutured with dissolvable stitches and skin flaps are sutured in double layer with an inverted-T scar in the same fashion as for cosmetic breast reduction.

The surgical markings of the Wise pattern are shown in Fig. 1a and are further described below.

A point (C’’) is marked along the inframammary fold (E–D) at a distance of 8–10 cm from the midsternal line. The breast meridian (dashed line) is marked as a vertical line passing from the midpoint of the clavicle (which is usually located 5 or 6 cm from the jugular notch) to C’’. The new position of the nipple-areola complex (A) is then marked along the breast meridian, at a distance of 19–22 cm from the jugular notch, in correspondence with the projection of the inframammary fold on the anterior surface of the breast. The two lateral margins of the marking are drawn by moving the breast laterally (Biesenberger/Aufricht maneuver) and drawing the medial line from the new site of the nipple to the point where the breast meridian crosses the inframammary sulcus (line B’-C’’) and, in the same way, by moving the breast medially and tracing the lateral line (lines B-C’’). Along the two lateral pillars (B-C’’ and B’-C’’), the distance from the lower border of the areola to the new inframammary sulcus is marked at 6 to 8 cm from points B and B’ (points C and C’). Two horizontal lines are drawn from points C and C’ to the inframammary sulcus (points E and D, respectively).

### Surgical technique

When indicated, axillary surgery (sentinel lymph node biopsy or axillary lymph node dissection) is performed first through a separate incision in the armpit.

At the beginning of the procedure, the nipple-areola complex (NAC) is outlined using a 38–45 mm cookie-cutter. Then, the WIBB flap is completely de-epithelized according to the preoperative markings of the Wise pattern, excluding the previously outlined nipple-areola complex and including the two triangular areas that are usually discharged in a routine reduction mammaplasty (CEC’’ and C’DC’’) (Fig. 1b).

The de-epithelialized area below the mosque dome is peripherally incised bilaterally along the vertical lines BC and B’C’ and the horizontal lines EC and C’D (double-incision technique) (Fig. 1c).

**Figure 2:**
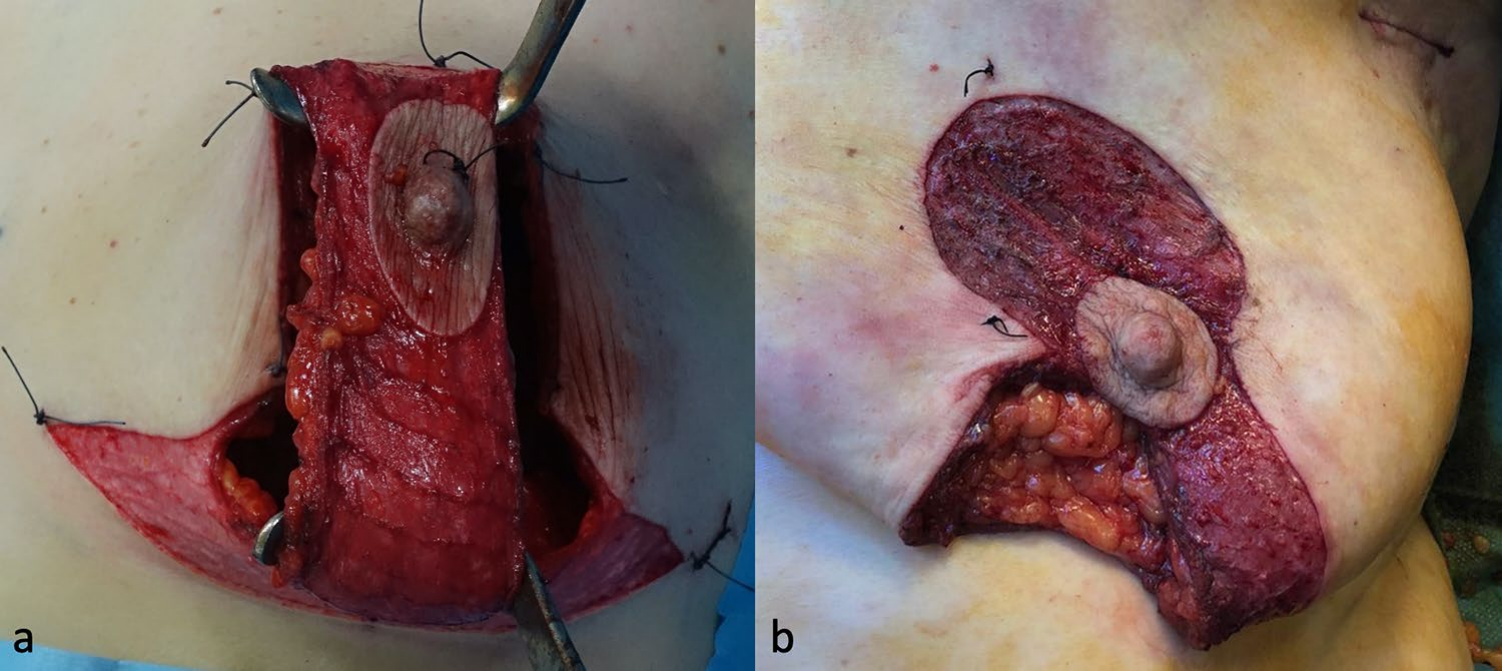
Intraoperative view of the elevated WIBB flap (**a**) Double-incision technique (**b**) Single-incision technique.

Mastectomy is performed through this wide access route with the nipple relying on the WIBB flap (Fig. 2a). Alternatively, the flap can be incised only along the medial or lateral aspect (e.g., only along the lines BC and EC) (single-incision technique) (Fig. 2b).

Reconstruction after NS/SRM can be immediate, with either autologous tissue or prepectoral implant and ADM (Fig. 1d), or otherwise staged, by using a temporary tissue expander (TE). The NAC is sutured in its new position upward (Fig. 1e), and skin flaps are then closed together in the typical inverted-T fashion (Fig. 1f).

Autologous or prosthetic reconstruction is agreed upon preoperatively with the patient; in the case of prosthetic reconstruction, the choice between immediate or two-stage reconstruction is often intraoperative, as discussed below.

### Reconstructive options

**Figure 3:**
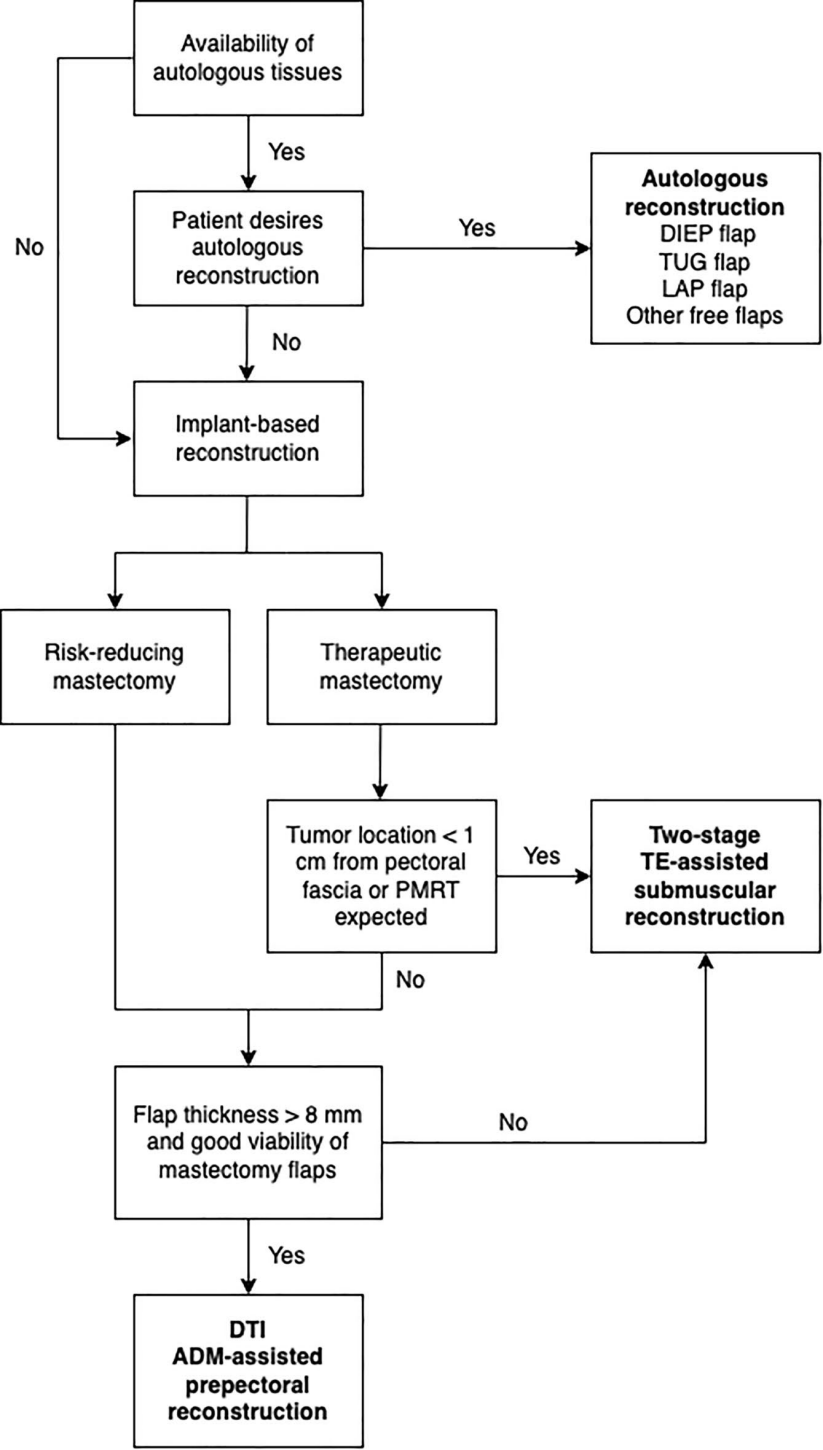
Decisional algorithm for breast reconstruction after NS/SRM (DIEP: Deep Inferior Epigastric Perforator; TUG: Transverse Upper Gracilis; LAP: Lumbar Artery Perforator; PMRT: Postmastectomy Radiation Therapy; TE: Tissue Expander; DTI: Direct-to-Implant; ADM: Acellular Dermal Matrix).

Our reconstructive algorithm takes into account the patient’s wishes and physical characteristics (e.g., availability of autologous tissues), the overall oncological aspects and position of the tumor, the potential adjuvant treatments if radiotherapy is planned, and the thickness and viability of the mastectomy flaps (Fig. 3).

#### Autologous reconstruction

When autologous reconstruction is planned, the DIEP flap becomes our first choice, as the targeted volume for reconstruction is in the range of 400–500 cc.

The WIBB flap described above allows for comfortable access to the recipient vessels—either the internal mammary or the subscapular axis—and flap insetting. The DIEP flap is completely de-epithelized and buried under the dermal flaps.

**Figure 4:**
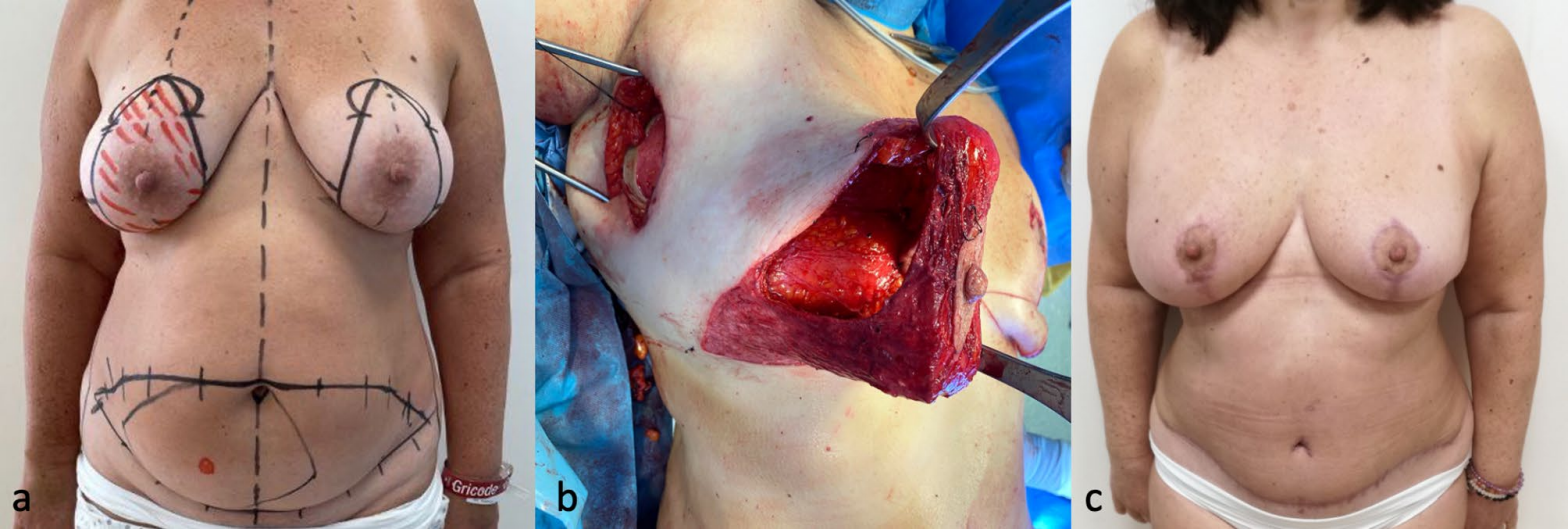
Fifty-two-year-old patient with right invasive ductal carcinoma who underwent NS/SRM, autologous reconstruction with DIEP flap, sentinel lymph node biopsy (SLNB) and contralateral symmetrization through breast reduction (**a**) Preoperative marking (**b**) DIEP flap buried under the WIBB flap before microsurgical anastomosis with subscapular vessels (**c**) Postoperative view at 3 months.

Flap monitoring is performed with a Doppler probe over the skin (Fig. 4).

#### Direct-to-implant (DTI) reconstruction

DTI reconstruction is preoperatively planned:

- After risk-reducing mastectomies (RRM)
- If no preoperative radiotherapy is planned
- If the tumor is more than one centimeter away from the pectoral fascia

We proceed with prepectoral reconstruction if the mastectomy flaps and the nipple-holding flap (WIBB flap) are at least 8 mm thick and appear well vascularized. Perfusion of mastectomy flaps is evaluated intraoperatively through clinical assessment of color, temperature, bleeding of incision edges and capillary refill. If perfusion is uncertain, skin viability is further evaluated through indocyanine green angiography and a photodynamic eye camera (ICG-PDE imaging system) (Hamamatsu Photonics K.K., Hamamatsu, Japan).

If the mastectomy flaps are thin or not well vascularized, we intraoperatively opt for a submuscular tissue expander.

In our series, the breast implant size fell within the range 380–600 cc; implants were placed in the prepectoral plane, directly under the WIBB flap and totally wrapped with an acellular dermal matrix (ADM).

**Figure 5:**
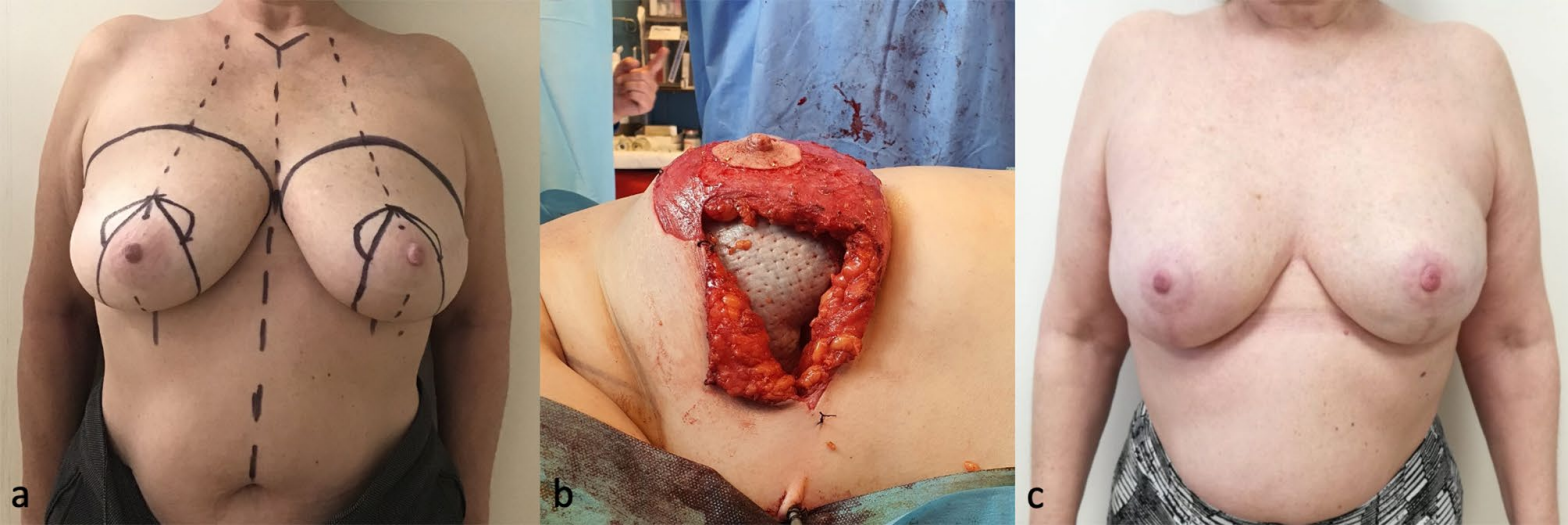
Fifty-seven-year-old patient with BRCA 1 gene mutation who underwent bilateral prophylactic mastectomy and immediate prepectoral reconstruction with implant and ADM (**a**) Preoperative marking (**b**) Intraoperative picture of de-epithelialized nipple-holding bipedicled dermal flap (WIBB flap) and prepectoral implant placement with complete ADM coverage (**c**) Postoperative picture 6 months after surgery.

The implant was therefore covered by a triple layer of tissue: skin, nipple-holding flap (WIBB flap) and ADM (Figs. 1d and 5).

#### Two-stage reconstruction

Two-stage reconstruction with a tissue expander is chosen intraoperatively:

- If mastectomy flaps are thinner than 8 mm, the expander reduces the compression on the mastectomy flaps and the NAC, thus reducing risk of skin necrosis
- For tumors located within 1 cm of the pectoral fascia, the patient is a candidate for adjuvant radiotherapy
- In case of simultaneous tissue expander positioning in the contralateral breast (delayed reconstruction after radical mastectomy in the contralateral breast)

The expander is placed entirely under the pectoralis major muscle and intraoperatively filled with a mean of 100 cc of saline; in these cases, the WIBB flap is placed over the muscular-fascial plane.

**Figure 6:**
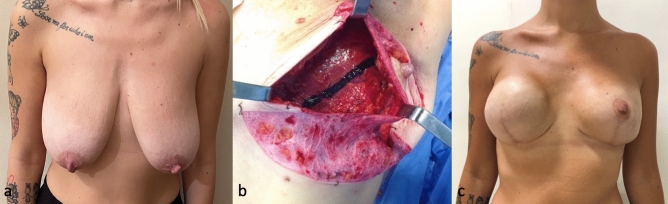
Thirty-three-year-old patient with bilateral multicentric carcinoma who underwent right SRM, left NS/SRM and submuscular reconstruction with breast expanders (**a**) Preoperative picture (**b**) Elevated WIBB flap and marking of the incision along the pectoralis major muscle (**c**) Postoperative picture at 6 months.

In the postoperative period, the expander is inflated progressively, in line with the recovery of skin flaps and avoiding excessive pressure. The expansion must be completed before beginning eventual postmastectomy radiation therapy (PMRT) (Fig. 6).

Regardless of the period needed to complete the expansion, the replacement of the expander with the definitive implant is deferred to at least six months after surgery or after completion of radiotherapy and is performed through an incision along the vertical scar or along the inframammary fold, as this delay is sufficient to effectively integrate the dermal flap.

### Cutaneous envelope

In all cases, immediate autologous, direct-to-implant or two-stage TE-assisted reconstruction is completed by suturing along the Wise pattern and the NAC (Fig. 1f).

## Results

Thirty-one patients underwent unilateral breast surgery (9 right and 22 left breasts), and 20 patients underwent bilateral breast surgery, totaling 71 operated breasts. The average age was 48.3 years (range 33–60, SD 8.6). BMI ranged from 21.5 to 30.9 (mean 24.8, SD 3.5). Fourteen patients had a BRCA gene mutation. No other pathogenic mutations predisposing patients to breast cancer were found in our cohort. The most common histological type requiring cancer removal was invasive ductal carcinoma (n = 32). No cancer was found in the nine specimens coming from the prophylactic mastectomies.

Fifty-four percent of patients (54.9%, n = 28) underwent two-stage reconstruction (40 breasts), and 45.1% (n = 23) underwent immediate reconstruction (31 breasts). Regarding the type of reconstruction, implant-based reconstruction was performed in most cases (45 patients, 65 breasts), and autologous reconstruction with a DIEP free flap was performed in 6 patients (6 unilateral reconstructions). For those who underwent immediate prepectoral breast reconstruction, ADMs were always used. The average implant size was 460 cc (range 380–600 cc, SD 47). The mean tissue expander size was 450 cc (range 400–600 cc, SD 48).

The average follow-up was 2.6 years (range 1–5, SD 1.2). Twenty-one percent of patients (n = 11) were smokers, 41.2% (n = 21) had comorbidities, and 31.4% (n = 16) underwent radiation therapy in association with a previous quadrantectomy (QUART). Three patients underwent neoadjuvant chemotherapy (5.9%), and 11 patients received adjuvant chemotherapy (21.6%). Adjuvant radiotherapy was performed in 35.3% of patients (n = 18). In 19.6% of cases (n = 10), patients underwent further surgical procedures (fat grafting, breast symmetrization).

Sixty-two mastectomies were therapeutic, while nine were performed for prophylaxis. In our series, most mastectomies (65 out of 71) were performed with the double-incision technique, while only 6 mastectomies were performed with a single-incision technique.

We only observed one major complication (1.4%), which was a periprosthetic infection that required implant removal. Minor complications occurred in 14.1% of cases (10 out of 71 breasts): we observed six cases of minor skin necrosis and two cases of partial NAC necrosis healed by secondary intention and two cases of seromas managed with drainage in the outpatient clinic. Data regarding sample characteristics are summarized in Tables 1 and 2.

**Table 1:** Characteristics of the patient sample.

Variable	*n* = 51
Age (mean ± SD, range) (years)	48.3 ± 8.6 (33–60)
BMI (mean ± SD, range) (kg/m^2^)	24.8 ± 3.5 (21.5–30.9)
Smoking (No, %)	
Yes	11 (21.6%)
No	40 (78.4%)
Comorbidities (No, %)	
Yes	21 (41.2%)
No	30 (58.8%)
Previous QUART* (No, %)	
Yes	16 (31.4%)
No	35 (68.6%)
Neoadjuvant chemotherapy	
Yes	3 (5.9%)
No	48 (94.1%)
Adjuvant radiation therapy	
Yes	18 (35.3%)
No	33 (64.7%)
Adjuvant chemotherapy	
Yes	11 (21.6%)
No	40 (78.4%)
Genetic mutation (No, %)	
BRCA1/2	14 (27.5%)
Other	0 (0.0%)
No	37 (72.5%)
Side of surgery (No, %)	
Right	9 (17.7%)
Left	22 (43.1%)
Bilateral	20 (39.2%)
Further surgical procedures	
Yes	10 (19.6%)
No	41 (80.4%)
Length of follow-up (mean ± SD, range) (years)	2.6 ± 1.2 (1–5)

*Quadrantectomy and radiation therapy.

**Table 2:** Characteristics of the 71 operated breasts.

Variable	*n* = 71
Intent of mastectomy	
Therapeutic	62 (87.3%)
Prophylactic	9 (12.7%)
Mastectomy approach	
Single incision	6 (8.5%)
Double incision	65 (91.5%)
Histological type (No, %)	
Invasive ductal carcinoma	32 (45.1%)
Invasive lobular carcinoma	17 (23.9%)
Mixed carcinoma	3 (4.2%)
Comedo-type ductal carcinoma	3 (4.2%)
Other types	7 (9.8%)
Timing of reconstruction (No, %)	
Two-stage	40 (56.3%)
Immediate	31 (43.7%)
Type of reconstruction (No, %)	
Implant-based	65 (91.5%)
Autologous	6 (8.5%)
Implant size (mean ± SD, range) (cc)	460 ± 47 (380–600)
Tissue expander size (mean ± SD, range) (cc)	450 ± 48 (400–600)
Overall complications (No, %)	11 (15.5%)
Minor	10 (14.1%)
Partial thickness skin necrosis	6 (8.5%)
Partial thickness NAC necrosis	2 (2.8%)
Periprosthetic seroma	2 (2.8%)
Major	1 (1.4%)
Infection with implant loss	1 (1.4%)

## Discussion

The WIBB flap is suitable for mastopexy and NS/SRM in cases of large and ptotic breasts. It provides broad surgical access to perform mastectomy and is suitable either for autologous or implant-based reconstruction.

Conservative mastectomies, mostly indicated for small breasts, are more difficult to perform on patients with medium-large and ptotic breasts due to both the possible complications related to the viability of the mastectomy skin flaps and the nipple-areola complex and to the remodeling of excess skin^13–20^. In this subset of patients, NS/SRM is considered the technique of choice^9,10^. Several approaches are currently available and mainly differ in terms of the surgical incision made to gain access to the mastectomy field, the type of nipple-bearing flap and the reconstructive indications^21–27^.

In this paper, we propose a modification of the mastectomy technique using a WideBase Bipedicled (WIBB) flap, which combines the principles of mastopexy with those of NS/SRM for cases of large and ptotic breasts. In 2015, Folli et al. reported a technical variation of NSM in which a bipedicled dermal flap that resembled the WIBB flap was used; however, some important differences can be highlighted: their flap was narrower because it did not include the “horizontal” limbs of the Wise pattern, it was always incised bilaterally, the vertical incisions started from the superior edge of the new areola and ended at the infra-mammary fold, and the technique was used only in association with submuscular tissue expander-assisted reconstruction^28^.

In our experience, the use of the WIBB flap showed a low incidence of major complications, such as necrosis of the nipple-areola complex and exposure or extrusion of the implant. Although a unilateral incision is also feasible, as we previously described, and could be considered a theoretically safer approach because the “horizontal” vascular supply to the WIBB flap is not interrupted bilaterally, we opted for this kind of “tripedicled" flap (superior, inferior and medial/lateral vascular supply) in only 6 cases, most of which were found in obese patients, and we expected a higher risk of complications; we were worried that a second incision could have jeopardized the vascular supply of the NAC^11^. Conversely, we usually preferred a double incision because it provides more comfortable access for the breast surgeon and allows the mobilization of the NAC more easily without apparently compromising its vascularization. In fact, our data analysis seems to show that the two techniques have a similar complication rate in terms of NAC or skin viability (10.8% in the double-incision technique vs. 16.7% in the single-incision technique). Although the difference between the two groups was not statistically significant, the paradoxically slightly lower complication rate in the double-incision subset could be explained by the lower amount of tension that is needed to move the NAC upward in its new position because the WIBB flap is detached bilaterally. Moreover, careful preoperative patient assignment to either the single- or the double-incision technique based on BMI evaluation may have played a role in this finding.

The decision to proceed with autologous or implant-based reconstruction depends on numerous individual and environmental factors. The reconstructive algorithm that we describe takes into consideration the oncological aspects, the potential adjuvant treatments and the patient’s willingness, comorbidities and physical characteristics. Preoperative consultation is key, conforming to the patient’s expectations.

In autologous reconstruction, the WIBB flap allows easy access to both the internal mammary and axillary recipient vessels as sites for anastomosis.

In implant-based reconstruction, the choice of immediate or two-stage reconstruction is made intraoperatively, in many cases, and represents a crucial point of our algorithm.

We prefer direct-to-implant reconstruction in prophylactic mastectomies if no preoperative radiotherapy has been administered preoperatively, if no vascular complications associated with the mastectomy flaps or the nipple-areola complex are expected and if the tumor is located more than 1 cm away from the pectoral fascia.

In our series of direct-to-implant reconstructions, the implant was always placed in the prepectoral plane and completely wrapped with an ADM.

In addition, the implants were covered with the WIBB flap. Hence, the technique allowed us to obtain a triple layer of coverage over the prosthesis (ADM, WIBB flap and skin), thus providing additional protection in the lower pole, where the weight of the implant can press and stretch the mastectomy flaps and prevent implant exposure in case of skin breakdown, especially at the T-junction^29^.

In fact, in case of partial necrosis of the skin flaps or the nipple, the implant remains protected by the dermal sling (the WIBB flap) and is not exposed; this allows the debridement of potential necrotic areas of the mastectomy flaps under local anesthesia without requiring implant removal or replacement. Another advantage of the WIBB flap is that it prevents the lower edge of the implant from being palpable, which is an unpleasant and frequent condition in implant reconstruction cases^30,31^.

Deciding on the correct implant volume when a change in size and envelope is expected, as in NS/SRM, has been largely discussed in the literature as a critical aspect^32,33^. Our approach allows the simple modification of the original breast size. We usually aim to achieve a C cup volume in the reconstructed breast, which is also the limit when using an ADM envelope. However, this can vary according to patient wishes.

Prepectoral reconstruction is beneficial for sparing the pectoralis major muscle without compromising the final cosmetic result. However, in some specific cases, such as in slim patients or when mastectomy flaps are too thin, ensuring sufficient implant coverage is difficult and high-risk. Postoperative radiotherapy could also affect prepectoral implant-based reconstruction, increasing the risk of capsular contracture and affecting the blood supply to mastectomy skin flaps^34–36^.

For these reasons, although immediate implant-based reconstruction is currently very popular, two-stage reconstruction represents 56% of cases in our series.

Preoperative planning of a tissue expander was performed for oncological reasons in cases of tumors located near the pectoral fascia (< 1 cm) that may require postoperative radiotherapy^37^ to avoid having the implant in the way of the radiation source; if the contralateral breast must be reconstructed with an expander, for example, due to previous radical mastectomy surgery (one case in our data); and if there are doubts concerning the viability of the mastectomy flaps or of the nipple that present intraoperatively. Particularly, the assessment of flap viability was conducted through clinical judgment of color, temperature, bleeding of incision edges and capillary refill. If perfusion was uncertain, indocyanine green angiography and a photodynamic eye camera (ICG-PDE imaging system) were employed as additional assessment tools before considering the patient not eligible for prepectoral reconstruction and planning a two-stage reconstruction. In fact, if compared to other tools such as thermal imaging and spectroscopy, ICG is still considered the best intraoperative mastectomy flap viability assessment method^38^.

The expander was always positioned in the submuscular plane and partially filled with saline intraoperatively. The use of a partially inflated expander decreased the tension on the breast skin and on the NAC, improving perfusion pressure and preventing mastectomy flap necrosis that could occur with the immediate use of implants^39–41^.

The expander was replaced with a definitive implant after completing expansions according to adjuvant treatments. The double vascularized layer between the mastectomy flaps and the implant—the WIBB flap and the pectoralis major muscle—effectively protects the implant from exposure without interfering with radiotherapy.

There is no agreement in the literature regarding postmastectomy radiotherapy timing for two-stage reconstruction. Ricci et al. reported an increased number of complications if post mastectomy radiotherapy is performed when an expander is in place^36^, while Oliver et al. and Ho et al. did not report an increased number of implant removals^42^ or a higher complication rate^43^ compared to radiotherapy with an expander or definitive implant in place.

In our case series, if PMRT was needed, patients received radiotherapy after completion of expansion, and replacement with a final implant was performed at least six months after completion of radiotherapy and approximately one year after mastectomy.

In our series, we had only one case of implant removal (major complication), no cases of total necrosis of the nipple-areola complex and eight cases of minor skin or NAC necrosis. The low complication rate observed in this series (1.4% of implant removal) indicates that the WIBB flap improves the NAC vascular supply and provides further implant coverage at the T-junction, allowing eventual healing by secondary intention and avoiding exposure and subsequent removal of the implant.

A limitation of this study is the small number of cases (71 reconstructed breasts), although the number of cases in all the previously published literature on NS/SRM is small; only Mosharrafa presents a series of 125 breasts with a 7% incidence of major complications requiring implant removal^44^.

## Conclusions

The proposed technique is a modification of routine NS/SRM in that a wide-base bipedicled (WIBB) flap is used. The flap presents many advantages: it provides easy access for mastectomy, it ensures better preservation of NAC perfusion, it provides additional coverage between the prosthesis and the skin, preventing implant exposure at the T-junction, and in the case of autologous reconstruction, it allows easy access to the recipient vessels.

Our study suggests that the application of this technique, in combination with the proposed reconstructive algorithm, significantly reduced the incidence of complications in our case series (especially major complications such as NAC necrosis and implant extrusion), ensuring oncological safety and a good cosmetic result at the same time.

## Conference presentation

The paper has been presented at the 32nd EURAPS Annual Meeting and at the 70th SICPRE Annual Meeting.

## Data Availability

The datasets generated and analyzed during the current study are available from the corresponding author upon reasonable request.
